# Tobacco smoke particles and indoor air quality (ToPIQ) - the protocol of a new study

**DOI:** 10.1186/1745-6673-6-35

**Published:** 2011-12-21

**Authors:** Daniel Mueller, Stefanie Uibel, Markus Braun, Doris Klingelhoefer, Masaya Takemura, David A Groneberg

**Affiliations:** 1 Department of Toxicology, Institute of Occupational Medicine, Social Medicine and Environmental Medicine, Goethe-University, Frankfurt, Germany

## Abstract

Environmental tobacco smoke (ETS) is a major contributor to indoor air pollution. Since decades it is well documented that ETS can be harmful to human health and causes premature death and disease. In comparison to the huge research on toxicological substances of ETS, less attention was paid on the concentration of indoor ETS-dependent particulate matter (PM). Especially, investigation that focuses on different tobacco products and their concentration of deeply into the airways depositing PM-fractions (PM10, PM2.5 and PM1) must be stated. The tobacco smoke particles and indoor air quality study (ToPIQS) will approach this issue by device supported generation of indoor ETS and simultaneously measurements of PM concentration by laser aerosol spectrometry. Primarily, the ToPIQ study will conduct a field research with focus on PM concentration of different tobacco products and within various microenvironments. It is planned to extend the analysis to basic research on influencing factors of ETS-dependent PM concentration.

## Introduction

The supply of clean air is regarded as one of the most important basic factors for the human health and wellbeing. In consequence, polluted air is able to threat human health and is considered as a major global health problem [1]. According to an estimation of the WHO (World Health Organization) approximately 2 million premature deaths worldwide per year are caused by air pollution [2]. Especially the quality of indoor air is of utmost importance for human health. Not only because people spend most of their time indoors (in industrialized countries, as the USA, up to almost 90 percent [3]) but also because the indoor concentration of pollutants is often much higher [4]. The wide range of indoor pollutants contains organic or inorganic chemicals, biological aerosols (bioaerosols) and particles. A major source of indoor air pollution is the environmental tobacco smoke (ETS, also called second hand smoke) [5-7], which is a mixture of exhaled mainstream smoke (MS) and sidestream smoke (SS) released from the smouldering tobacco product. Since decades it is well documented that ETS can be harmful to human health and causes premature death and disease to the non-smoking population [8]. Especially ETS exposed children have an increased risk for acute respiratory infections, sudden infant death syndrome, more severe asthma and ear problems [6,8]. In the adult population, exposure to ETS is associated with acute coronary heart disease [9-11] and lung cancer [12,13]. According to a 2004 published estimation by Öberg et al., almost half of the world's children (approx. 40%) are regularly exposed to ETS followed by nonsmoking women (35%) and men (33%) [14]. Although exposure to ETS appears to present smaller risks than active smoking, the large percentage of exposed people, coupled with evidences that ETS causes illness and premature death, demonstrates a substantial public health threat. Because of these adverse effects to human health, tobacco smoke has been intensely investigated. To date, about 5000 individual compounds have been quantitatively determined in cigarette smoke [15], including many toxic substances as well as 69 carcinogens, of which 11 are known human carcinogens and 7 are probably carcinogenic in humans [16]. Many of these toxic and carcinogenic substances can be found in ETS as well. Particulate matter (PM) is one of those harmful components that can be found in ETS and is responsible for ETS as a substantial contributor to the level of particulate indoor air pollution [17]. Because of their capability to deposit deeply in the respiratory tract, particles of the PM10- and PM2.5-fraction can cause serious health problems. For a long time, PM10 and PM2.5 have been proven to be associated with acute and chronic health effects. Epidemiological data suggesting that exposure to particle pollution (PM10 and PM25) is able to increase morbidity and mortality of cardiopulmonary diseases like pre-existing COPD [18-23], cardiovascular diseases [24-26], exacerbation of asthma [20,27,28] and other conditions [29]. In addition, exposure to PM and especially to PM2.5 has been linked to the development of cancer [30]. However, the exact mechanism of cancer induction due to PM is still not resolved. Regarding the impact of both ETS and PM on human health, only few data is published about the concentration of PM in ETS so far.

## Aims

It is the aim of the ToPIQ study to assess the particle concentrations (PM10, PM2.5 and smaller particle fractions) that are produced by different tobacco products under a multitude of different conditions. Next to the determination of ETS-dependent PM concentrations within various microenvironments, like vehicle cabins, this study aims to examine the role of physical influencing factors on the PM concentration.

## Methods

**Figure 1:**
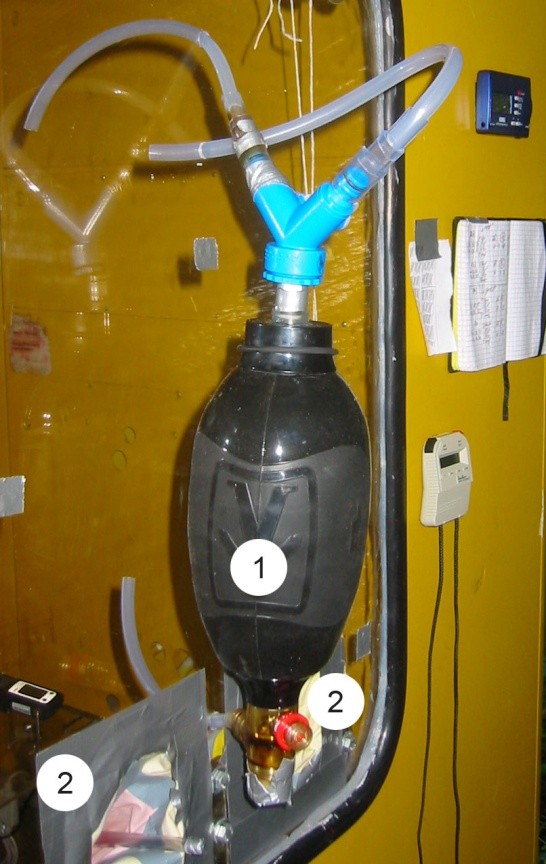
**Picture taken from outside of the testing chamber (telephone cabin) showing the ETSE (1) and the glove ports (2)**.

For the implementation of the ToPIQ study (ToPIQS), generation of ETS it will be necessary. To avoid health risks on human smokers a self-made ETS emitter (ETSE) will be used for the indoor ETS generation (Figure 1). Basically, the ETSE consists of a bag valve mask (BVM) plus tubing by which MS from the burning cigarette can be collected and afterwards vented out into the testing chamber. Throughout the experiment the burning tobacco product will be situated inside the testing chamber, producing the SS in between the time of MS collection. When the bag is inflating it collects the smoke inside. During the compression of the bag, the smoke will be released in the chamber. The compression and decompression of the bag will follow a predefined protocol under support of acoustic signals. The hand-operating ETSE will be attached outside of the chamber. There, the researcher can operate the device without the potential harm of an ETS exposure. Glove ports on the outside of the chamber will provide an isolated access to the chamber (Figure 1). In the future, the implementation of an automatic ETSE (AETSE) in the study is planned. With this device, simulations of ETS emitted by multiple smokers will be conducted.

**Figure 2:**
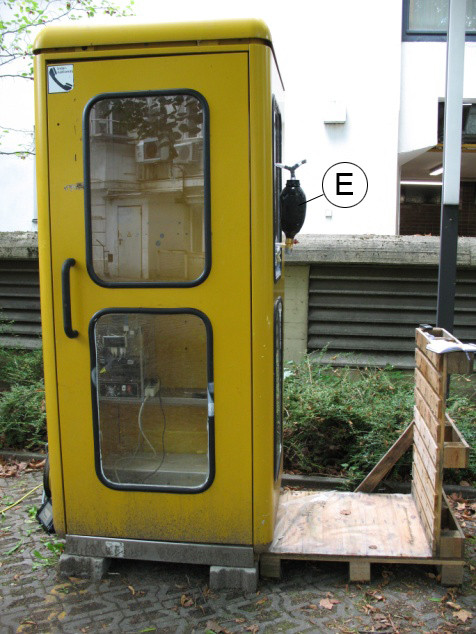
**Testing chamber (telephone cabin) with outside mounted ETSE (E)**.

The experiments will be carried out in different microenvironments. For the basic research on ETS of different tobacco types, a 1.75 m^3 ^telephone cabin will be used as an ETS test chamber (Figure 2). To simulate natural conditions the test chamber will be placed on an outdoor area in urban surrounding. Inside the chamber, mobile sensing modules will be placed, which will continuously measure the concentration of particulate matter (PM10, PM2.5 and PM1) and physical parameters (temperature, humidity, wind velocity). Subsequently, the measured data will be saved on an ultra-mobile PC unit. The generation of indoor ETS will be performed by the ETSE. Monitoring will be carried out in different ventilation modes with open and closed windows. In a next step, basic studies on the effect of volume size will be conducted on self-constructed testing chambers with a sizes-range of some cubic centimeters to several cubic meters. To study the effect of physical parameters on ETS particle concentration, the environmental conditions in these chambers will be kept stable. In future setups it is planned to investigate microenvironments of various vehicle cabins. Similar to the procedure at the testing cabin, mobile sensing modules will be monitoring PM and physical parameters inside the vehicle during ETS generation by the ETSE. The measurements will take place in stationary cars with focus on the effect of different window positions and different air vent or air-conditioning modes on the particle concentration of PM10, PM2.5 and PM1. To study the influence of different driving conditions on the particle concentration, they will be simulated in stationary cars with the help of ventilators. In each testing chamber, the mobile sensing module will be mounted inside the chamber at a location where children are potentially exposed to ETS. To create comparable settings, all conditions (ETS generation, test chamber, measurement) will be standardized. Prior to every sequence, a 20-minute background collection of the PM-concentration will be performed. Since a part of the sampling will be taken outdoors, it is important to prevent data bias due to environmental factors. Therefore, the measurements of the different tobacco products or cigarette brands will be measured alternately with a reference cigarette. To avoid bias due to daily variation of PM concentration, a data correction will be performed. For that reason, values of the prior collected average PM background concentration will be subtracted from the measured data during and after the ETS emission.

Initially, the received basic data from the measurements will be processed. Each average background concentration will then be subtracted from the concentration of the following ETS measurement to avoid bias from daily variation of PM concentration. The obtained data of the different sensors will be integrated. Subsequently, the data of every measurement will be divided in the two intervals "ETS emission" and "ETS elimination". The interval "ETS emission" will represent the phase of ETS generation and the interval "ETS emission" will outline the time where the ETS concentration will be reduced due to processes of ventilation and deposition. For both intervals and for every PM-fraction the arithmetic mean (C_mean_-PM), the maximum concentration (C_max_-PM), and the area under the curve (AUC-PM) will be calculated. Following data processing, an exploratory data analysis will be carried out. Data processing and analysis will be performed using specific calculating and statistical software.

## Discussion

So far, large scale assessment of PM generation by tobacco products was not performed. Therefore, only little data is available in scientific databases such as PubMed, Medline or ISI-Web. Novel approaches including scientometric and visualizing techniques are not applicable [31-43] and the few existing studies can easily be summarized. Early researches of particulate matter concentration in ETS focused on respirable suspended particle mass (RSP) [44-47]. Distinction between different PM-fraction (PM10, PM2.5, and PM1) and cigarette brands, as planned in the ToPIQ study, however, were not made. Since two of these published articles were conducted or supported by cigarette companies [44,45] the impartiality of these results is at least debatable. In most of these studies the ETS generation was carried out by human smokers in special testing chambers with a capacity of 18 to 45 m^2 ^[44,45,47]. Although realistic ETS generation can be guaranteed by using human smokers, this approach is dangerous to human health and therefore unethical. That is why an ETSE or AETSE will be used in the ToPIQ study. Other studies undertaken in the last decade focused on the ETS-dependent emissions of PM10 or PM2.5 or both [48-53], but of these studies only three investigated specific cigarette brands as planned in the ToPIQ study [48,50,51]. Only two of these studies were performed without human smokers by using smouldering cigarettes [48,51]. However, the usage of smouldering cigarettes for the ETS generation is insufficient since smouldering cigarettes can only produce SS and no MS. That is why the emissions generated by this method are not comparable to ETS emissions. To simulate ETS for the research in the ToPIQ study, we will use ETSE or AETSE, which are capable of generating SS as well as MS and therefore the two major components of ETS.

## Conclusion

The ToPIQ study will serve as a new platform to investigate ETS-dependent particulate matter of different tobacco products and within variable microenvironments. Using the knowledge of this platform, further studies may focus on mechanisms by which particulate matter harms the human body, i.e. with the use of modern techniques of toxicology [54,55], molecular biology [56-60] and biochemistry [61-64].

## List of abbreviations

AETSE: automatic environmental tobacco smoke emitter; AUC-PM: area under the PM-concentration curve; BVM: bag valve mask; C_max_-PM: maximum PM-concentration; C_mean_-PM: arithmetic mean of the PM-concentration; ETS: environmental tobacco smoke; ETSE: environmental tobacco smoke emitter; MS: mainstream smoke; PM: particulate matter; RSP: respirable suspended particle mass; SS: sidestream smoke; ToPIQS: Tobacco smoke particles and indoor air quality study; WHO: World Health Organization.

## Competing interests

The authors declare that they have no competing interests.

## Authors' contributions

DM, SU, MB, DK, SB, MS, DAG have made substantial contributions to the conception and design of the review, acquisition of the review data and have been involved in drafting and revising the manuscript. All authors have read and approved the final manuscript.

## References

[B1] Groneberg DA Morfeld P Kraus T Kohler D Krug N Magnussen H Nowak D Rabe KF Schultze-Werninghaus G Schulz H Health effects of particulate matter exposure: current scientific knowledge Pneumologie (Stuttgart, Germany) 2009 63 363 368 10.1055/s-0029-1214788 19591081

[B2] Air Quality And Health-WHO Fact Sheet No. 313 http://www.who.int/mediacentre/factsheets/fs313/en/

[B3] Klepeis NE Nelson WC Ott WR Robinson JP Tsang AM Switzer P Behar JV Hern SC Engelmann WH The National Human Activity Pattern Survey (NHAPS): a resource for assessing exposure to environmental pollutants J Expo Anal Environ Epidemiol 2001 11 231 252 10.1038/sj.jea.7500165 11477521

[B4] The American Lung Association (ALA), The Environmental Protection Agency (EPA), The Consumer Product Safety Commission (CPSC), (AMA) TAMA Indoor Air Pollution: An Introduction for Health Professionals 1994 New York, Washington, D.C, Chicago, IL: American Lung Association, Environmental Protection Agency, Consumer Product Safety Commission, American Medical Association

[B5] Repace JL Lowrey AH Indoor air-pollution, tobacco-smoke, and public-health Science 1980 208 464 472 10.1126/science.7367873 7367873

[B6] Tobacco Free Initiative International Consultation on EnvironmentalTobacco Smoke (ETS) and Child Health Book International Consultation on EnvironmentalTobacco Smoke (ETS) and Child Health. Editor ed.^eds 1999 City: World Health Organisation (WHO) 1 29

[B7] Nazaroff WW Singer BC Inhalation of hazardous air pollutants from environmental tobacco smoke in US residences J Expo Anal Environ Epidemiol 2004 14 S71 S77 15118748 10.1038/sj.jea.7500361

[B8] U.S. Department of Health and Human Services The health consequences of invountary exposure to tobacco smoke: A report of the Surgeon General Book The health consequences of invountary exposure to tobacco smoke: A report of the Surgeon General. Editor ed.^eds 2006 29 City: U.S. Department of Health and Human Services 1 27

[B9] He Y Lam TH Li LS Du RY Jia GL Huang JY Zheng JS Passive smoking at work as a risk factor for coronary heart-disease in chinese women who have never smoked Br Med J 1994 308 380 384 10.1136/bmj.308.6925.380 PMC2539443 8124145

[B10] Pell JP Haw S Cobbe S Newby DE Pell ACH Fischbacher C McConnachie A Pringle S Murdoch D Dunn F Smoke-free legislation and hospitalizations for acute coronary syndrome N Engl J Med 2008 359 482 491 10.1056/NEJMsa0706740 18669427

[B11] Glantz SA Parmley WW Passive smoking and heart-disease-Mechanisms and risk Jama-Journal of the American Medical Association 1995 273 1047 1053 10.1001/jama.1995.03520370089043 7897790

[B12] World Health Organisation (WHO) Tobacco Smoke and Involuntary Smoking IARC Monographs on the Evaluation of Carcinogenic Risk to Humans 2004 83 PMC4781536 15285078

[B13] Hackshaw AK Law MR Wald NJ The accumulated evidence on lung cancer and environmental tobacco smoke Br Med J 1997 315 980 988 10.1136/bmj.315.7114.980 PMC2127653 9365295

[B14] Öberg M Jaakkola MS Woodward A Peruga A Prüss-Ustün A Worldwide burden of disease from exposure to second-hand smoke: a retrospective analysis of data from 192 countries The Lancet 2011 377 139 146 10.1016/S0140-6736(10)61388-8 21112082

[B15] Prefontaine D Morin A Jumarie C Porter A In vitro bioactivity of combustion products from 12 tobacco constituents Food Chem Toxicol 2006 44 724 738 10.1016/j.fct.2005.10.005 16324776

[B16] Hoffmann D Hoffmann I The Changing Cigarette: Chemical Studies and Bioassays Book The Changing Cigarette: Chemical Studies and Bioassays. Editor ed.^eds 2001 City: U.S. Department of Health and Human Services

[B17] United States. Public Health Service The health consequences of involuntary smoking: a report of the Surgeon General Book The health consequences of involuntary smoking: a report of the Surgeon General. Editor ed.^eds 1986 City: United States. Public Health Service. Office on Smoking and Health

[B18] Atkinson RW Anderson HR Sunyer J Ayres J Baccini M Vonk JM Boumghar A Forastiere F Forsberg B Touloumi G Acute effects of particulate air pollution on respiratory admissions-Results from APHEA 2 project Am J Respir Crit Care Med 2001 164 1860 1866 11734437 10.1164/ajrccm.164.10.2010138

[B19] Ko FWS Hui DSC Outdoor air pollution: impact on chronic obstructive pulmonary disease patients Curr Opin Pulm Med 2009 15 150 157 10.1097/MCP.0b013e32832185ee 19532031

[B20] Dominici F Peng RD Bell ML Pham L McDermott A Zeger SL Samet JM Fine particulate air pollution and hospital admission for cardiovascular and respiratory diseases Jama-Journal of the American Medical Association 2006 295 1127 1134 10.1001/jama.295.10.1127 PMC3543154 16522832

[B21] Sunyer J Basagana X Particles, and not gases, are associated with the risk of death in patients with chronic obstructive pulmonary disease Int J Epidemiol 2001 30 1138 1140 10.1093/ije/30.5.1138 11689536

[B22] Ghio AJ Sint T Donohue JF Ambient air pollution particles and the acute exacerbation of chronic obstructive pulmonary disease Inhal Toxicol 2008 20 25 29 10.1080/08958370701758759 18236218

[B23] Pope CA Kanner RE Acute effects of PM-10 pollution on pulmonary-function of smokers with mild-to-moderate chronic obstructive pulmonary-disease Am Rev Respir Dis 1993 147 1336 1340 8503541 10.1164/ajrccm/147.6_Pt_1.1336

[B24] Vidale S Bonanomi A Guidotti M Arnaboldi M Sterzi R Air pollution positively correlates with daily stroke admission and in hospital mortality: a study in the urban area of Como, Italy Neurol Sci 2010 31 179 182 20119741 10.1007/s10072-009-0206-8

[B25] Gehring U Heinrich J Kramer U Grote V Hochadel M Sugiri D Kraft M Rauchfuss K Eberwein HG Wichmann HE Long-term exposure to ambient air pollution and cardiopulmonary mortality in women Epidemiology 2006 17 545 551 10.1097/01.ede.0000224541.38258.87 16755270

[B26] Brook RD Rajagopalan S Pope CA Brook JR Bhatnagar A Diez-Roux AV Holguin F Hong YL Luepker RV Mittleman MA Particulate Matter Air Pollution and Cardiovascular Disease An Update to the Scientific Statement From the American Heart Association Circulation 2010 121 2331 2378 10.1161/CIR.0b013e3181dbece1 20458016

[B27] Tecer LH Alagha O Karaca F Tuncel G Eldes N Particulate matter (PM2.5, PM10-2.5, and PM10) and children's hospital admissions for asthma and respiratory diseases: A bidirectional case-crossover study Journal of Toxicology and Environmental Health-Part a-Current Issues 2008 71 512 520 10.1080/15287390801907459 18338286

[B28] Gavett SH Koren HS The role of particulate matter in exacerbation of atopic asthma Int Arch Allergy Immunol 2001 124 109 112 10.1159/000053685 11306943

[B29] Yeatts K Svendsen E Creason J Alexis N Herbst M Scott J Kupper L Williams R Neas L Cascio W Coarse particulate matter (PM2.5-10) affects heart rate variability, blood lipids, and circulating eosinophils in adults with asthma Environ Health Perspect 2007 115 709 714 10.1289/ehp.9499 17520057 PMC1867980

[B30] Pope CA Burnett RT Thun MJ Calle EE Krewski D Ito K Thurston GD Lung Cancer, Cardiopulmonary Mortality, and Long-term Exposure to Fine Particulate Air Pollution JAMA: The Journal of the American Medical Association 2002 287 1132 1141 10.1001/jama.287.9.1132 PMC4037163 11879110

[B31] Groneberg-Kloft B Fischer TC Quarcoo D Scutaru C New quality and quantity indices in science (NewQIS): the study protocol of an international project J Occup Med Toxicol 2009 4 16 10.1186/1745-6673-4-16 19555514 PMC2708171

[B32] Vitzthum K Scutaru C Musial-Bright L Quarcoo D Welte T Spallek M Groneberg-Kloft B Scientometric analysis and combined density-equalizing mapping of environmental tobacco smoke (ETS) research PLoS One 2010 5 e11254 10.1371/journal.pone.0011254 20582305 PMC2889821

[B33] Groneberg-Kloft B Kreiter C Welte T Fischer A Quarcoo D Scutaru C Interfield dysbalances in research input and output benchmarking: visualisation by density equalizing procedures International journal of health geographics 2008 7 48 10.1186/1476-072X-7-48 18724868 PMC2533656

[B34] Scutaru C Quarcoo D Takemura M Welte T Fischer TC Groneberg-Kloft B Density-equalizing mapping and scientometric benchmarking in Industrial Health Ind Health 2010 48 197 203 10.2486/indhealth.48.197 20424350

[B35] Groneberg-Kloft B Quarcoo D Scutaru C Quality and quantity indices in science: use of visualization tools EMBO Rep 2009 10 800 803 19648952 10.1038/embor.2009.162 PMC2726685

[B36] Scutaru C Quarcoo D Sakr M Shami A Al-Mutawakel K Vitzthum K Fischer TC Zuberbier T Groneberg-Kloft B Density-equalizing mapping and scientometric benchmarking of European allergy research J Occup Med Toxicol 2010 5 2 10.1186/1745-6673-5-2 20925908 PMC2843702

[B37] Groneberg-Kloft B Scutaru C Dinh QT Welte T Chung KF Fischer A Quarcoo D Inter-disease comparison of research quantity and quality: bronchial asthma and chronic obstructive pulmonary disease J Asthma 2009 46 147 152 10.1080/02770900802503115 19253120

[B38] Kusma B Scutaru C Quarcoo D Welte T Fischer TC Groneberg-Kloft B Tobacco control: visualisation of research activity using density-equalizing mapping and scientometric benchmarking procedures Int J Environ Res Public Health 2009 6 1856 1869 10.3390/ijerph6061856 19578464 PMC2705221

[B39] Groneberg-Kloft B Scutaru C Fischer A Welte T Kreiter C Quarcoo D Analysis of research output parameters: density equalizing mapping and citation trend analysis BMC Health Serv Res 2009 9 16 10.1186/1472-6963-9-16 19171075 PMC2672943

[B40] Borger JA Neye N Scutaru C Kreiter C Puk C Fischer TC Groneberg-Kloft B Models of asthma: density-equalizing mapping and output benchmarking J Occup Med Toxicol 2008 3 Suppl 1 S7 10.1186/1745-6673-3-S1-S7 18315838 PMC2259401

[B41] Groneberg-Kloft B Scutaru C Kreiter C Kolzow S Fischer A Quarcoo D Institutional operating figures in basic and applied sciences: Scientometric analysis of quantitative output benchmarking Health research policy and systems/BioMed Central 2008 6 6 18554379 10.1186/1478-4505-6-6 PMC2459159

[B42] Zell H Quarcoo D Scutaru C Vitzthum K Uibel S Schoffel N Mache S Groneberg DA Spallek MF Air pollution research: visualization of research activity using density-equalizing mapping and scientometric benchmarking procedures J Occup Med Toxicol 2010 5 5 10.1186/1745-6673-5-5 20359334 PMC2865481

[B43] Vitzthum K Scutaru C Quarcoo D Mache S Groneberg DA Schoffel N Cardiac insufficiency: a critical analysis of the current publication procedures under quantitative and qualitative aspects J Cardiothorac Vasc Anesth 2010 24 731 734 10.1053/j.jvca.2009.05.010 19632859

[B44] Martin P Heavner DL Nelson PR Maiolo KC Risner CH Simmons PS Morgan WT Ogden MW Environmental tobacco smoke (ETS): A market cigarette study Environment International 1997 23 75 90 10.1016/S0160-4120(96)00079-7

[B45] Nelson PR Conrad FW Kelly SP Maiolo KC Richardson JD Ogden MW Composition of environmental tobacco smoke (ETS) from international cigarettes and determination of ETS-RSP: Particulate marker ratios Environment International 1997 23 47 52 10.1016/S0160-4120(96)00076-1

[B46] Phillips K Howard DA Browne D Lewsley JM Assessment of personal exposures to environmental tobacco-smoke in British nonsmokers Environment International 1994 20 693 712 10.1016/0160-4120(94)90303-4

[B47] Leaderer BP Hammond SK Evaluation of vapor-phase nicotine and respirable suspended particle mass as markers for environmental tobacco-smoke Environ Sci Technol 1991 25 770 777 10.1021/es00016a023

[B48] Invernizzi G Ruprecht A Mazza R Rossetti E Sasco A Nardini S Boffi R Particulate matter from tobacco versus diesel car exhaust: an educational perspective Tob Control 2004 13 219 221 10.1136/tc.2003.005975 15333875 PMC1747905

[B49] Invernizzi G Ruprecht A De Marco C Mazza R Tagliapietra L Michieletto F Allegri F Sbrogio L Bettoncelli G Sasco A Boffi R Smoking in car: monitoring pollution of particulate matter as mass and as particle number, of organic volatile compounds and of carbon monoxide. Evaluating the most suitable ETS marker, and the effect of opening the driver's Window Epidemiology 2011 22 194

[B50] Maziak W Rastam S Ibrahim I Ward KD Eissenberg T Waterpipe-associated particulate matter emissions Nicotine Tob Res 2008 10 519 523 10.1080/14622200801901989 18324571

[B51] Ott W Klepeis N Switzer P Air change rates of motor vehicles and in-vehicle pollutant concentrations from secondhand smoke J Expo Sci Environ Epidemiol 2008 18 312 325 10.1038/sj.jes.7500601 17637707

[B52] Rees VW Connolly GN Measuring air quality to protect children from secondhand smoke in cars Am J Prev Med 2006 31 363 368 10.1016/j.amepre.2006.07.021 17046406

[B53] Vardavas CI Linardakis M Kafatos AG Environmental tobacco smoke exposure in motor vehicles: a preliminary study Tob Control 2006 15 415 415 16998180 10.1136/tc.2006.017459 PMC2563645

[B54] Groneberg DA Grosse-Siestrup C Fischer A In vitro models to study hepatotoxicity Toxicol Pathol 2002 30 394 399 10.1080/01926230252929972 12051557

[B55] Eynott PR Groneberg DA Caramori G Adcock IM Donnelly LE Kharitonov S Barnes PJ Chung KF Role of nitric oxide in allergic inflammation and bronchial hyperresponsiveness Eur J Pharmacol 2002 452 123 133 10.1016/S0014-2999(02)02237-9 12323393

[B56] Groneberg DA Doring F Nickolaus M Daniel H Fischer A Expression of PEPT2 peptide transporter mRNA and protein in glial cells of rat dorsal root ganglia Neurosci Lett 2001 304 181 184 10.1016/S0304-3940(01)01794-3 11343832

[B57] Dinh QT Groneberg DA Peiser C Mingomataj E Joachim RA Witt C Arck PC Klapp BF Fischer A Substance P expression in TRPV1 and trkA-positive dorsal root ganglion neurons innervating the mouse lung Respir Physiol Neurobiol 2004 144 15 24 10.1016/j.resp.2004.08.001 15522699

[B58] Lauenstein HD Quarcoo D Plappert L Schleh C Nassimi M Pilzner C Rochlitzer S Brabet P Welte T Hoymann HG Pituitary adenylate cyclase-activating peptide receptor 1 mediates anti-inflammatory effects in allergic airway inflammation in mice Clin Exp Allergy 2011 41 592 601 10.1111/j.1365-2222.2010.03636.x 21059121 PMC3694437

[B59] Dinh QT Groneberg DA Peiser C Springer J Joachim RA Arck PC Klapp BF Fischer A Nerve growth factor-induced substance P in capsaicin-insensitive vagal neurons innervating the lower mouse airway Clin Exp Allergy 2004 34 1474 1479 10.1111/j.1365-2222.2004.02066.x 15347383

[B60] Groneberg DA Fischer A Chung KF Daniel H Molecular mechanisms of pulmonary peptidomimetic drug and peptide transport Am J Respir Cell Mol Biol 2004 30 251 260 14969997 10.1165/rcmb.2003-0315TR

[B61] Eynott PR Paavolainen N Groneberg DA Noble A Salmon M Nath P Leung SY Chung KF Role of nitric oxide in chronic allergen-induced airway cell proliferation and inflammation J Pharmacol Exp Ther 2003 304 22 29 10.1124/jpet.102.040295 12490571

[B62] Groneberg DA Bester C Grutzkau A Serowka F Fischer A Henz BM Welker P Mast cells and vasculature in atopic dermatitis--potential stimulus of neoangiogenesis Allergy 2005 60 90 97 10.1111/j.1398-9995.2004.00628.x 15575937

[B63] Peiser C Springer J Groneberg DA McGregor GP Fischer A Lang RE Leptin receptor expression in nodose ganglion cells projecting to the rat gastric fundus Neurosci Lett 2002 320 41 44 10.1016/S0304-3940(02)00023-X 11849759

[B64] Groneberg DA Peiser C Dinh QT Matthias J Eynott PR Heppt W Carlstedt I Witt C Fischer A Chung KF Distribution of respiratory mucin proteins in human nasal mucosa Laryngoscope 2003 113 520 524 10.1097/00005537-200303000-00023 12616207

